# Boosting the Energetic Performance of Trinitromethyl-1,2,4-oxadiazole Moiety by Increasing Nitrogen-Oxygen in the Bridge

**DOI:** 10.3390/ijms231710002

**Published:** 2022-09-02

**Authors:** Peng Chen, Hui Dou, Chunlin He, Siping Pang

**Affiliations:** 1 School of Materials Science & Engineering, Beijing Institute of Technology, Beijing 100871, China; 2 Experimental Center of Advanced Materials, School of Materials Science & Engineering, Beijing Institute of Technology, Beijing 100081, China; 3 Yangtze Delta Region Academy of Beijing Institute of Technology, Jiaxing 314019, China; 4 Chongqing Innovation Center, Beijing Institute of Technology, Chongqing 401120, China

**Keywords:** energetic materials, trinitromethyl moieties, furazan, 1,2,4-oxadiazole, azo group, detonation performance, sensitivity

## Abstract

The trinitromethyl moiety is a useful group for the design and development of novel energetic compounds with high nitrogen and oxygen content. In this work, by using an improved nitration method, the dinitromethyl precursor was successfully nitrated to the trinitromethyl product (**2**), and its structure was thoroughly characterized by FTIR, NMR, elemental analysis, differential scanning calorimetry, and single-crystal X-ray diffraction. Compound **2** has a high density (1.897 g cm^−3^), high heat of formation (984.8 kJ mmol^−1^), and a high detonation performance (*D*: 9351 m s^−1^, *P*: 37.46 GPa) that may find useful applications in the field of high energy density materials.

## 1. Introduction

The goal for designing and synthesizing novel energetic compounds is to achieve new materials with higher performance, better safety, and improved environmental compatibility [1,2]. The trinitromethyl group has been found to be a versatile functional group for constructing high energy density materials (HEDMs) and energetic oxidizers [3,4]. This moiety is useful to improve the overall performance of an energetic compound by enhancing the density and oxygen balance [5,6]. Several methods have been used to synthesize trinitromethyl-based heterocyclic compounds by nitrating dinitromethyl, ethyl acetate, or an acetonyl group [7,8,9,10]. Subsequently, several trinitromethyl heterocyclic compounds with excellent properties were developed based on those methods [11,12,13,14].

**Figure 1:**
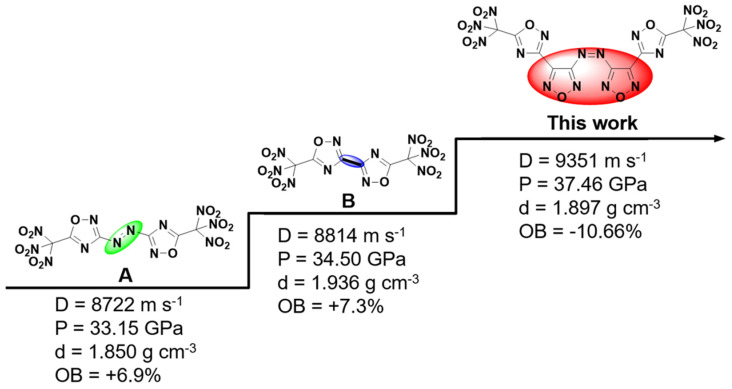
Performance comparison of compound **2** with two compounds **A** and **B** containing the 5-trinitromethyl-1,2,4-oxadiazole group.

Oxadiazoles, five-membered heterocycles with high nitrogen and oxygen content and high heats of formation, were selected to design and construct a large number of high-performance energetic compounds by incorporating with the trinitromethyl group [15,16,17]. In 2014, Klapötke’s group reported the synthesis of 5,5′-bis(trinitromethyl)-3,3′bi(1,2,4-oxadiazole) (**B**), which exhibited high density, good detonation performance, and positive oxygen balance and showed great potential as energetic oxidizers (Figure 1) [18]. By replacing the C-C bridge in compound **B** with an azo bridge, 5,5′-bis(trinitromethyl)-3,3′-azo-1,2,4-oxadiazole (**A**) was synthesized by Shreeve’s group [19], with the help of the azo group, the thermal stability for **A** was slightly improved and the heat of formation was dramatically increased compared to that of **B**. Among the four oxadiazole isomers, 1,2,5-oxadiazole (furazan 216.3 kJ mol^−1^) had a higher heat of formation than 1,2,4-oxadiazole (99.8 kJ mol^−1^). Therefore, with a fixed number and type of substituted groups, the replacement of 1,2,4-oxadiazole backbone with furazan always resulted in better properties [20]. In this work, a novel energetic compound 4,4′-(5-trinitromethyl-1,2,4-oxadiazole)-3,3′-azo-furazan (**2**) was designed and synthesized by incorporating azo-furazan with 5-trinitromethyl-1,2,4-oxadiazole moieties. The predicted density of compound **2** reached 1.967 g cm^−3^ by using the molecular surface electrostatic potentials method based on Gaussian 09 calculation and Multiwfn 3.7 [21]. In view of the high predicted density and the high heat of formation, we believe that the detonation performance of the trinitromethyl derivative **2** will be greatly improved and would show great potential as a high explosive.

## 2. Results and Discussion

### 2.1. Synthesis

**Scheme 1:**
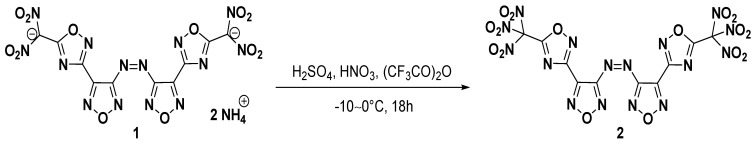
Synthetic procedures for preparing compound **2**.

The precursor, diammonium 4,4′-(5-dinitromethyl-1,2,4-oxadiazole)-3,3′-azo-furazanate **1**, was prepared according to the method in the literature [22]. Our initial attempt to prepare compound **2** by nitrating compound **1** using the mixture of fuming nitric acid and concentrated sulfuric acid according to the procedure similar to the literature failed [17,18,19]. However, when a small amount of trifluoroacetic anhydride was added to the mixed acids, compound 1 was successfully nitrated to give 4,4′-(5-trinitromethyl-1,2,4-oxadiazole)-3,3′-azo-furazan (**2**) by maintaining the temperature at −5 °C for 18 h, resulting in a yield of 86.67%. The role of trifluoroacetic anhydride in the reaction system may be to disperse the *gem*-dinitromethyl compound generated in the reaction to ensure that the nitration process can proceed smoothly. The orange solid **2** was formed and isolated by pouring the reaction mixture onto ice followed by filtration (Scheme 1).

### 2.2. ^15^N Multinuclear Magnetic Resonance

Compound **2** was fully characterized by Fourier Transform Infrared (FTIR) (Figure S4), multinuclear magnetic resonance (NMR) spectroscopy (Figure S1), differential scanning calorimetry (DSC) (Figure S3), and elemental analysis. In the ^15^N NMR spectrum (Figure S2), there were six signals; nitrogen atoms of the trinitromethyl and azo bridge were found at δ = +142.4 ppm (N1) and δ = −40.3 ppm (N6), while the nitrogen atoms in the 1,2,4-oxidazole ring were found at δ = −116.6 ppm (N4) and δ = −0.8 ppm (N5), and the nitrogen atoms in the furazan ring were found at δ = 50.4 ppm (N2) and δ = 37.2 ppm (N3), respectively.

### 2.3. Single-Crystal X-ray Analysis

**Figure 2:**
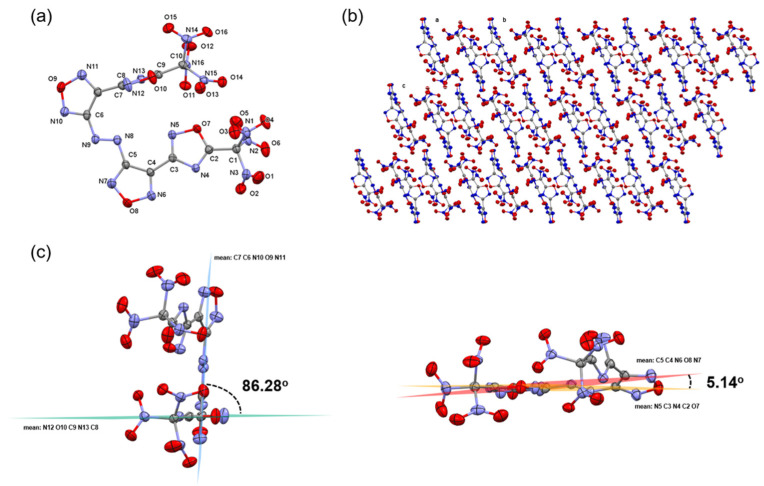
(**a**) Crystal structure of **2**; (**b**) packing diagram of **2**; (**c**) the angle between the furoxan plane and the 1,2,4-oxadiazole ring plane on both sides of the azo group, respectively.

Suitable crystals of **2** (CCDC† 2182869) were obtained by recrystallization from a solution of trifluoroacetic acid (Figure 2), which crystallized in the triclinic space group *P*-1 had a calculated density of 1.908 g cm^−3^ at 170 K with two molecules per unit cell (detailed crystallographic data in Table S1). The two furoxan ring and azo group were not coplanar, which was proven by the torsion angles C7−C6−N9−N8 (−0.67°) and N7−C5−N8−N9 (−24.567°). Compound **2** had mixing *π* − *π* stacking, and no significant intermolecular interactions were found between the two conformational molecular layers in Figure 2b. As shown in Figure 2c, the angles between the planes of the adjacent furazan ring and the 1,2,4-oxadiazole ring were 86.28° and 5.14°, respectively. The trinitromethyl groups on the 1,2,4-oxidazole ring were arranged in tetrahedral geometry, which was similar to the compounds reported in the literature [23].

## 3. Physicochemical and Energetic Properties

### 3.1. Hirshfeld Surface

**Figure 3:**
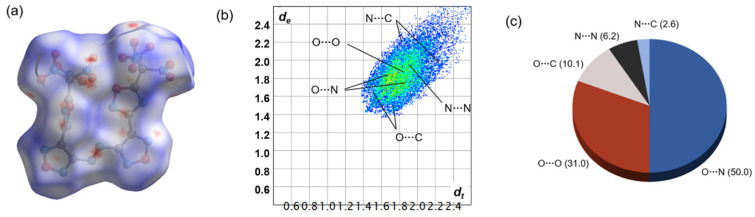
(**a**) Hirshfeld surfaces in the crystal stacking of **2**. (**b**) 2D fingerprint plot of **2**. (**c**) The pie graph of **2**, which shows the percent contributions of the individual atomic contacts to the Hirshfeld surface.

Trinitromethyl derivative **2** contains a high amount of oxygen and nitrogen (N + O = 80.0%), which is detrimental to its sensitivity. To understand the relationship between the structural and physical properties, two-dimensional (2D)-fingerprint spectra and Hirshfeld electrostatic surface plots were analyzed systematically using CrystalExplorer 3.1 (Figure 3) [24]. The red and blue dots on the Hirshfeld surface analysis represent high and low close contact populations, respectively, in Figure 3a. The red dots indicate strong intermolecular (O…O and O…N) interactions, and the relative contribution of the contacts are reflected in regular 2D fingerprint plots in Figure 3b. The major interactions were O…O (31.0%) and O…N (50.0%) and the absence of hydrogen bonding interactions in **2**, suggesting relatively sensitive properties for compound **2**.

### 3.2. Density, Differential Scanning Calorimetry, and Oxygen Balance

Density is one of the most important factors in determining the performance of energetic compounds. The densities of **2** were measured using an AccuPyc II 1345 gas pycnometer (25 °C). The combination of furazan, 1,2,4-oxadiazole, and the trinitromethyl groups gave the molecule a high density of 1.897 g cm^−3^ (Table 1). The thermal properties of compound **2** were analyzed using DSC; it had a melting point at 58.1 °C and an onset decomposition temperature at 115.9 °C. Its low melting temperature may be attributed to absence of hydrogen atoms in the structure, which make it difficult to form hydrogen bonds in the structure. The higher energy for **2** also resulted in a lower thermal stability compared to that of **A** (125 °C) and **B** (124 °C). More carbon atoms in the structure gave compound **2** a negative oxygen balance of −10.66%.

### 3.3. Heats of Formation, Sensitivity, and Detonation Performance

The heats of formation (Δ*H_f_*) were calculated by employing the Gaussian 09 (Revision E.01) suite of programs [25] and the isodesmic reactions method (Scheme S1), as shown in the Supplementary Materials (Tables S2 and S3). The results are listed in Table 1. Benefiting from the high enthalpy of furazan-1,2,4-oxadiazole backbone, compound **2** exhibited a high positive heat of formation of 984.8 kJ mol^−1^, which was much higher than that of compounds **A** (461.4 kJ mol^−1^) and **B** (61.9 kJ mol^−1^). The sensitivities toward friction and impact for **2** were measured by using standard BAM methods. The trinitromethyl group in the structure led to highly sensitive properties towards both impact and friction (*IS*: 3 J, *FS*: 40 N) for compound **2**. Subsequently, the detonation performances of **2** were calculated by employing the EXPLO5 (v6.05) program using the measured densities and the calculated solid-state heats of formation [26]. As show in Table 1, the physiochemical properties of compounds **2** were compared with **A**, **B**, and conventional explosives RDX and HMX. The detonation properties of **2** (*D*: 9351 m s^−1^, *P*: 37.46 GPa) were a lot better than the azo bridge linked trinitromethyl compounds **A** (*D*: 8722 m s^−1^, *P*: 33.15 GPa) and C-C bridge linked compound **B** (*D*: 8814 m s^−1^, *P*: 34.50 GPa), and the detonation velocity of **2** was even better than HMX (*D*: 9144 m s^−1^). Its high detonation performances make it a potential candidate as a high explosive.

## 4. Experimental Section

Caution! All the nitrogen-rich compounds used are energetic materials and may explode under certain conditions. Appropriate safety precautions should be taken when preparing and/or handling. Only small quantities should be prepared and studied.

### 4.1. General Methods

^1^H and ^13^C spectra were recorded using a 400 MHz nuclear magnetic resonance spectrometer Bruker AVANCE NEO 400 at a frequency of 400.13 MHz and 100.62 MHz, respectively. The chemical shifts in the ^13^C NMR spectra were reported relative to Me_4_Si. The melting and decomposition points were obtained at a heating rate of 5 °C min^−1^ and a flow rate of dry nitrogen gas of 50 mL min^−1^ using a Discovery 25 differential scanning calorimeter from TA Instruments Co. IR spectra were recorded using a Thermoscientific Summit PRO FT-IR in KBr pellets that aim at solids. Densities were determined at 25 °C by employing a Micromeritics AccuPycII 1345 gas pycnometer. Elemental analyses were carried out using a Thermo Scientific FLASH 2000 elemental analyzer. The impact and friction sensitivities were measured using a standard BAM fall hammer and a BAM friction tester, respectively.

### 4.2. Synthesis of Compound 4,4′-(5-Trinitromethyl-1,2,4-Oxadiazole)-3,3′-Azo-Furazan (***2***)

Compound **1** was prepared according to the method in the literature [22]. The reaction of 3- amino-4-aminoximinofurazan with methyl malonyl chloride gave 3-amino-4-(5-acetoxymethyl-1,2,4-oxadiazole)furazan, followed by reacting with concentrated hydrochloric acid and potassium permanganate to achieve an azo compound 4,4′-(5-Methyl dinitroacetate-1,2,4-oxadiazole)-3,3′-azofurazan. Subsequently, the azo compound was nitrated by mixed acid and then reacted with ammonia to obtain compound **1**. Compound **1** (0.27 g, 0.5 mmol) was dissolved in concentrated sulfuric acid (3.0 mL) and cooled to −10 °C. Fuming nitric acid (1.5 mL) was added dropwise to the reaction mixture over 30 min. The temperature of this reaction was kept below 0 °C during this addition. After that, trifluoroacetic anhydride (1 mL) was added at one time to the reaction mixture and stirred at −5 °C for 18 h. An orange precipitate formed, after which the reaction mixture was poured into ice (20.0 g). The precipitate was collected by filtration, washed with cold water (5.0 mL), and dried at room temperature to give a pure orange solid compound **2** (0.26 g, 86.67%). *T_m_*: 58.1 °C, *T_dec_*: 115.9 °C; IR (cm^−1^) ν = 1605, 1565, 1504, 1469, 1438, 1272, 1143, 1080, 1030, 980, 925, 900, 867, 840, 796, 768, 746, 704, 683, 626, 593, 522, 508, 450, 405; ^13^C NMR (Acetone-d6, TMS): δ 118.8, 139.4, 139.6, 160.7, 163.3, 163.8; ^15^N NMR (Acetone-d6): −116.6, −40.3, −0.8, 37.2, 50.4, 142.4. EA (C_10_N_16_O_16_, 600.21): calculated (%): C 20.01, N 37.34; found: C 19.97, N 37.32.

## 5. Conclusions

In summary, a novel trinitromethyl compound **2** was successfully synthesized using an improved nitration method with a yield of 86.67%. Compound **2** has a high density (1.897 g cm^−3^) and an onset decomposition temperature (115.9 °C), which is lower than that of **A** (125 °C) and **B** (124 °C). It also exhibits high mechanical insensitivity (*IS*: 3 J, *FS*: 40 N). The properties of **2** were interpreted and discussed by both experimental and theoretical calculations. Its superior detonation properties (*D*: 9351 m s^−1^, *P*: 37.46 GPa) and oxygen balances to those of HMX (*D*: 9144 m s^−1^, *P*: 39.2 GPa) makes it an attractive candidate as a new HEDM.

## Figures and Tables

**Table 1:** Physicochemical and energetic properties of compounds **2**, **A,** and **B** compared with those of RDX and HMX.

Compd	*T_m_^a^* /°C	*T_d_^b^* /°C	*ρ ^c^* /g cm^−3^	Δ*H_f_^d^* /kJ mol^−1^	*D ^e^* /m s^−1^	*P ^f^* /GPa	*IS ^g^* /J	*FS ^h^* /N	OB *^i^* /%
2	58.1	115.9	1.897	984.766	9351	37.46	3	40	−10.66
A	-	125	1.850	461.4	8722	33.15	5	80	+6.9
B	-	124	1.936	61.9	8814	34.50	10	80	+7.3
RDX *^j^*	-	204	1.80	70.3	8795	34.9	7.5	120	−21.62
HMX *^j^*	-	287	1.91	74.8	9144	39.2	7.4	120	−21.62

*^a^* Melting temperature (onset temperature at heating rate of 5 °C min^−1^). *^b^* Decomposition temperature (onset temperature at heating rate of 5 °C min^−1^). *^c^* Density, measured—gas pycnometer (25 °C). *^d^* Heat of formation calculated using Gaussian 09 (Revision E.01). *^e^* Detonation velocity –calculated using EXPLO5 V6.05. *^f^* Detonation pressure –calculated using EXPL O5 V6.05. *^g^* Impact sensitivity. *^h^* Friction sensitivity. *^i^* Oxygen balances for C_a_H_b_O_c_N_d_, 1600 (c–2a–b/2)/MW; MW = molecular weight—based on CO_2_ formation. *^j^* Ref [27].

## Data Availability

Data can be found in Supplementary Materials.

## Supplementary Materials

The supporting information can be downloaded at: https://www.mdpi.com/article/10.3390/ijms231710002/s1.
