# Supplementary material for: Boosting the Energetic Performance of Trinitromethyl-1,2,4-oxadiazole Moiety by Increasing Nitrogen-Oxygen in the Bridge
Source: Int J Mol Sci. 2022 Sep 2;23(17):10002. doi: 10.3390/ijms231710002 (PMC9456194; doi:10.3390/ijms231710002)
Supplement: Supplementary file 1 [file ijms-23-10002-s001.zip › ijms-1887086-supplementary.pdf]

Supplementary Materials

Boosting the Energetic Performance of Trinitrome-thyl-1,2,4-oxadiazole Moiety by Increasing Nitrogen-Oxygen in the Bridge

Peng Chen,[1,2] Hui Dou,[1,2] Chunlin He,[1,2,3,4]* and Siping Pang [1,]*

1 School of Materials Science & Engineering, Beijing Institute of Technology, Beijing 10081, China
2 Experimental Center of Advanced Materials, School of Materials Science & Engineering, Beijing Institute of Technology, Beijing 10081, China.;
3 Yangtze Delta Region Academy of Beijing Institute of Technology, Jiaxing 314019, China.
4 Chongqing Innovation Center, Beijing Institute of Technology, Chongqing 401120, China.
* Correspondence: chunlinhe@bit.edu.cn (C.H.); pangsp@bit.edu.cn (S.P.)

Table of Contents

Crystal data and structure refinement 1

^{13}C NMR and ^{15}N NMR spectra 2

Differential scanning calorimetry (DSC) scans 3

Fourier Transform Infrared (FT-IR) 3

Gaussian Calculation 4

Crystal data and structure refinement

Table S1 Crystal data and structure refinement for **2**.

Identification code	**2**
CCDC number	2182869
Empirical formula	$C_{10}N_{16}O_{16}$
Formula weight	600.26
Temperature/K	170
Crystal system	triclinic
Space group	*P*-1
a/Å	9.8874(8)
b/Å	10.4237(10)
c/Å	11.3885(9)
α/°	116.162(3)
β/°	95.794(3)
γ/°	91.412(4)
Volume/Å^3	1044.96(16)
Z	2
ρ_{calc}g/cm^3	1.908
μ/mm^{-1}	0.182
F(000)	600.0
Crystal size/mm^3	0.08 × 0.06 × 0.04
Radiation	MoKα (λ = 0.71073)
2Θ range for data collection/°	4.016 to 52.956
Index ranges	$-12 \leq h \leq 11$, $-12 \leq k \leq 13$, $-14 \leq l \leq 14$
Reflections collected	12351
Independent reflections	4289 [R_{int} = 0.0715, R_{sigma} = 0.0782]
Data/restraints/parameters	4289/0/379
Goodness-of-fit on F^2	1.047
Final R indexes [I>=2σ (I)]	R_1 = 0.0463, wR_2 = 0.1003
Final R indexes [all data]	R_1 = 0.0783, wR_2 = 0.1197
Largest diff. peak/hole / e Å^{-3}	0.26/-0.31

^{13}C NMR and ^{15}N NMR spectra

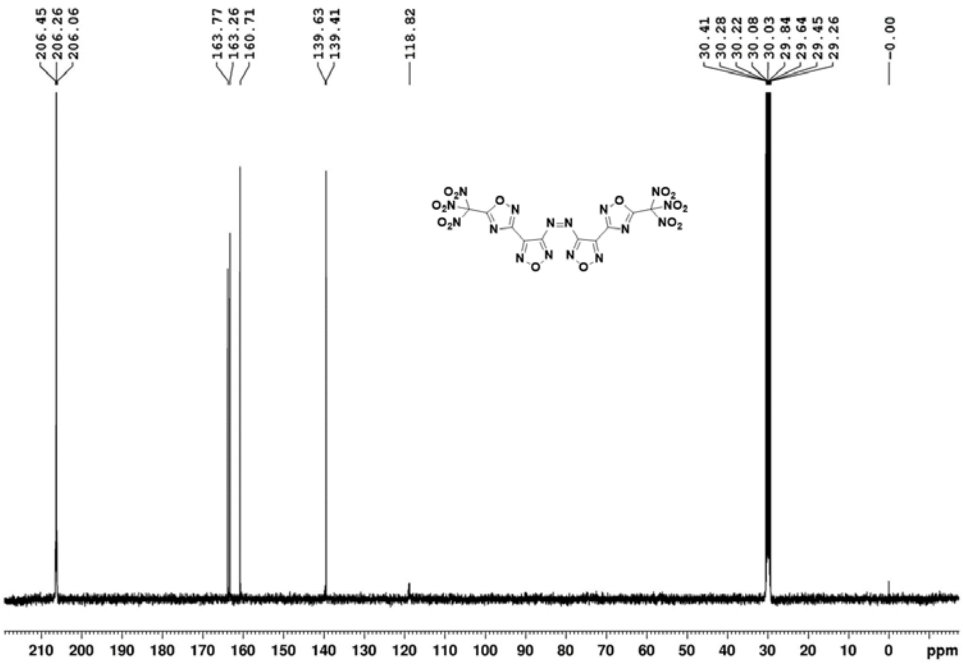

Figure S1. ^{13}C NMR of **2**.

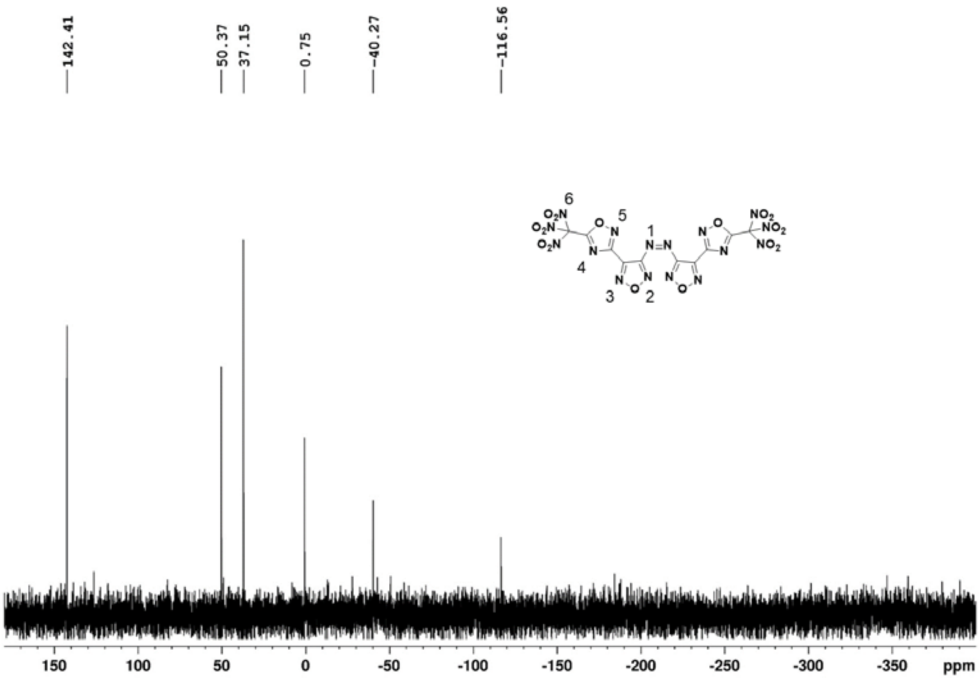

Figure S2. ^{15}N NMR of **2**.

Differential scanning calorimetry (DSC) scans

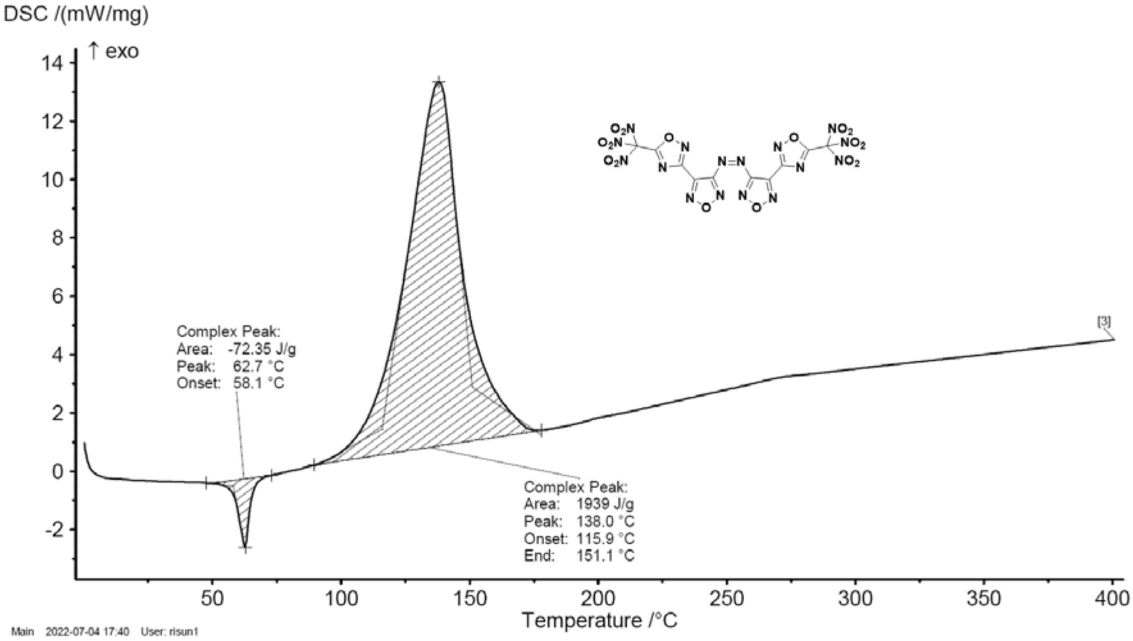

Figure S3. DSC curves of 2 under nitrogen with a heating rate of 5 °C min^{-1}

Fourier Transform Infrared (FT-IR)

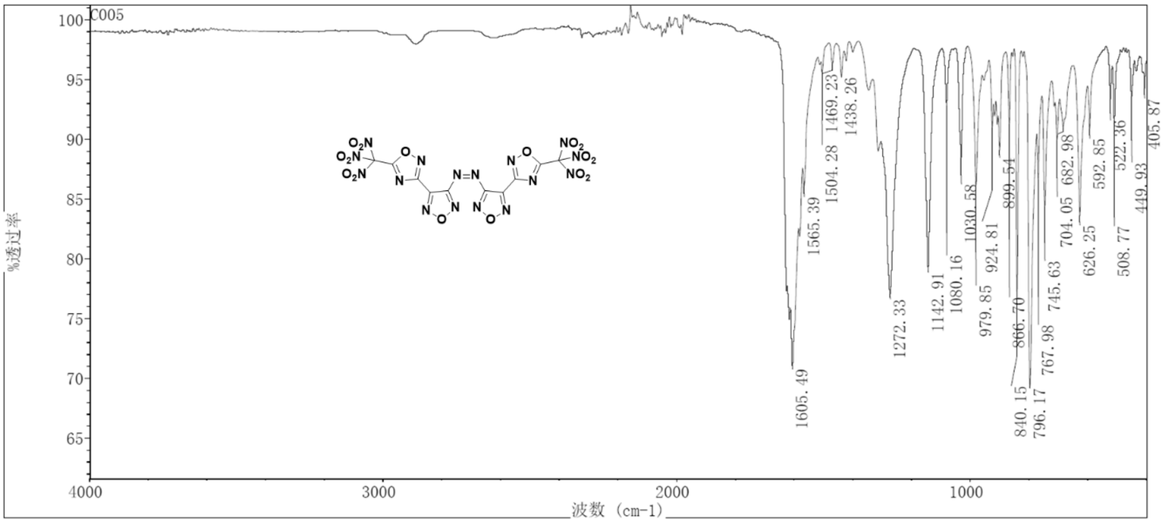

Figure S4. FT-IR curves of 2

Gaussian Calculation

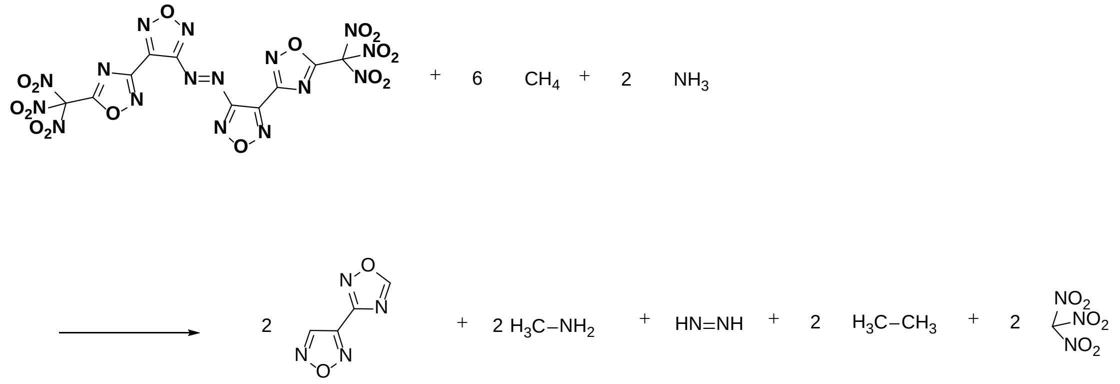

Scheme S1. Isodesmic reactions for calculating heats of formation for the anions of **2.**

Table S2. Calculated (B3LYP-D3BJ/6-31+G(d,p) // M062x/def2QZVPP) total energy(E_0), zero-point energy (*ZPE*), values of the correction (H_r), and heats of formation (HOF) for anions.

	ZPE	Hr	E_0	Corrected E_0	HOF (KJ mol^{-1})
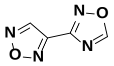	0.072807	0.080642	-522.9863084	-522.90858	306.17[b]
CH_3CH_3	0.074962	0.079382	-79.8143982	-79.73801	-84[a]
N_2H_2	0.028522	0.032322	110.6471796	-110.61600	200.41[b]
CH_3NH_2	0.064305	0.068654	-95.8543147	-95.78823	-23[a]
CH_4	0.045014	0.048823	-40.5062133	-40.45919	-74.6[a]
NH_3	0.034517	0.038327	-56.5578458	-56.52090	-45.9[a]
Nitrofrom	0.054646	0.064483	-654.0296587	-653.96736	-27.74 [b]
**2**	0.201407	0.237123	-2459.86609	-2459.63702	1047.07 [b]

[a] Data from NIST, [b] Calculated from G2.

Table S3. Calculation of Heat of formation by Atomization G2

	E_{298} (G2)	MW	D_0 (Hartree)	ΔH_f^{298} (Kcal/mol)	ΔH_f^{298} (KJ/mol)
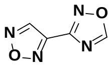	-522.2	138.0844	2.022031	73.17633821	306.1697991
N_2H_2	-110.48	30.02936	0.440344	47.89995673	200.413419
Nitrofrom	-653.18	151.0353	1.448821	-6.6289413	-27.7354904

Solid-state heats of formation of the resulting compound **2** were calculated with Equation (S1) in which T_{dec} is the decomposition temperature.

$$\mathbf{\Delta Hf = \Delta Hf(g) - \Delta Hsub = \Delta Hf(g) - 188[J\ mol^{-1}\ K^{-1}] \times Tm} \cdots\cdots\cdots\cdots \text{(S1)}$$
